# Successful spinal anaesthesia for caesarean section in an African patient with Takayasu’s arteritis

**DOI:** 10.11604/pamj.2018.30.281.16182

**Published:** 2018-08-20

**Authors:** Mamo Woldu Kassa, Tadele Melese Benti, Alemayehu Ginbo Bedada

**Affiliations:** 1 Department of Anaesthesia and Critical Care, University of Botswana, Botswana; 2 Department of Obstetrics and Gynaecology, University of Botswana, Botswana; 3 Department of Surgery, University of Botswana, Botswana

**Keywords:** African patient, caesarean section, spinal anaesthesia, Takayasu´s arteritis

## Abstract

Takayasu's arteritis (TA) is a rare chronic inflammatory disease affecting mainly the aorta and its main branches. We report a case of a 24-year-old primigravida, an African patient, with TA planned for caesarean section at 37 weeks of gestation. Clinically, she has involvement of aortic arch and its branches and abdominal aorta. She underwent caesarean section and delivered an alive baby boy under successful spinal anaesthesia with insignificant complications. Although it is rare in the African continent, anesthesiologists should be up-to-date with the knowledge of perioperative anesthetic management of TA in pregnant cases requiring operative delivery.

## Introduction

Takayasu's arteritis (TA) is a rare chronic progressive inflammatory disease of uncertain aetiology, but suspected to have multiple causes [1-11]. Takayasu first described the disease in 1908 [1-8]. TA usually affects aorta and its main branches commonly: carotid, subclavian and renal arteries [1-10]. TA affects more women than men with a ratio ranging from 4:1to 8:1[2-4, 10, 11]. The peak incidence is in the 2^nd^-3^rd^ decades [5]. It is common in Japan [1], Asia and South America, and less common in Europe and North America [4-6, 10]. Intimal infiltration by lymphocytes and other inflammatory cells results in the replacement of vessel wall elastic tissue by fibrous tissue with subsequent formation of stenosis, occlusion and aneurysm [1, 5]. The major cause of hypertension in TA is renovascular but it can result from an abnormal function of the carotid and aortic sinus baroreceptors and/or reduced elasticity and a marked narrowing of the aorta and major arteries [8]. Clinical symptoms depend on the distribution of the involved vasculature and ischemic disturbance of the organs affected. This may result in claudication, ischemic pain and fatigue of the limbs, and carotid arteries may give headache, vertigo, syncope, convulsions, transient hemiplegia, aphasia, and visual disturbance; renal artery involvement may cause hypertension and some patients may progress to aortic insufficiency and congestive heart failure [1, 2, 5, 9]. Physical findings depends on the affected artery and include high blood pressure and reduction or loss of palpable pulses in the neck and limbs [7, 9]. Pulmonary involvement leads to pulmonary hypertension [9]. Management of TA involves corticosteroid and other immunosuppressive agents [1, 5, 10, 11]. Some cases of TA may require further treatment in the form of angioplasty or surgical correction [4, 5, 7, 10]. The commonest cause of death in TA are heart failure, myocardial infarction (MI) and stroke [5, 11]. Fatal complications during pregnancy include aortic aneurysm rupture and cerebral haemorrhage [10]. Anaesthesia in TA is complicated by uncontrolled hypertension leading to end organ dysfunction, stenosis of major blood vessels affecting regional circulation, and difficulties in the monitoring of arterial blood pressure [8]. The initial manifestation of TA may occur during pregnancy [4]. The effect of pregnancy on TA is unclear [1, 5, 7, 9]. But in 60-90% of cases hypertensive complications including preeclampsia, exacerbated chronic hypertension, miscarriage or fetal loss are reported [5-7]. Reports on spinal anaesthesia for caesarean section for the management of patients with TA in African continent is almost non-existent. We are presenting a case of TA pregnant patient who successfully delivered by caesarean section using spinal anaesthesia.

## Patient and observation

We are presenting a 24-year-old African premegravida who was diagnosed with Takayasu's arteritis and underwent caesarean section under spinal anesthesia at Princess Marina Hospital, the largest tertiary teaching hospital in Gaborone, Botswana. She is 37 weeks pregnant weighing 65kg and 160cm of height. She was diagnosed with TA 5 years ago fulfilling four of the American college of rheumatology diagnostic criteria: age < 40 years, blood pressure difference of more than 10mmHg in right and left arm, claudication and bruit in the abdominal aorta. She was scheduled for elective caesarean section for severe oligohydramnios with intrauterine growth restriction (IUGR). She was under treatment for her TA and hypertension taking amlodipine 10mg peros per day, methyldopa 500mg twice peros per day and prednisolone 5mg peros per day. Except for occasional headache the hypertension and TA were well controlled. On examination she looks healthy, with BP 140/80mmHg on her right arm and 190/101mmHg on her left arm with pulse rate of 96 per minute. All pulses in both lower limb are present. She has no features of cardiac failure. Laboratory tests such as: full blood count, serum electrolyte, and renal function tests were normal. Echocardiogram showed 60% left ventricular ejection fraction and mild left ventricular hypertrophy and normal right ventricle. Preoperative anaesthetic evaluation showed oxygen saturation of 98% on room air, Mallampatti airway and ASA each class 2. Peripheral venous excess was secured on the left forearm and 500ml of lactated ringer was infused to overcome acute reduction of blood pressure following spinal anaesthesia. She was given amoxicillin 2gm Peros, ranitidine 50mg Intravenous, metoclopramide injection 10mg Intravenous, and hydrocortisone 100mg Intravenous to prevent Addison's crisis.

She was positioned in sitting position and a 25G quinicke's spinal needle is used to administer 1.5ml of 0.5% hyperbaric bupivacaine combined with 20μg fentanyl at L3-L4 space under aseptic condition. Sensory block was achieved at T4 level. A pillow was placed under her neck to prevent stretching of the carotid arteries with further compromise in blood flow, tilted to the left lateral to prevent aorto-caval compression. She was monitored with three lead electrocardiogram, pulse oximeter on her left index finger, and non-invasive blood pressure. Following administration of the spinal anaesthesia there was a drop of BP in the right arm from 140/80mmHg to 120/60mmHg which was corrected back to base line by 1l of lactated ringer. No vasopressor was used as the blood pressure remained in the range of 140/80mmHg to 135/78mmHg. Lower segment caesarean section was performed and a baby boy with APGAR score of 9 and 10 at 5^th^ and 10^th^ minute weighing 2.2kg was delivered. Then 10 IU of oxytocin in 500ml of lactated ringer was infused post-delivery. The operation took 25 minutes and the estimated blood loss was 500ml. Patient was admitted to post-natal high dependency ward. Postoperatively her blood pressure remained around preoperative levels. She was discharged home on fifth postoperative day on her regular PO medications and advised to follow at postnatal and cardiology clinic.

## Discussion

Takayasu's arteritis is a rare disease usually affecting aorta and its main branches [1-10]. Majority are women [2-4, 10, 11], with pick incidence in second and third decades [5] as the case in our patient. It is common in Japan, [1] Asia and South America, and less common in Europe and North America [4-6, 10]. Although TA is reported in Africa, reports in association with pregnancy and spinal anesthesia is limited. American college of Rheumatology diagnostic criteria for TA: age of onset 40 or less, claudication, aortic or subclavian bruits, decreases brachial pulses, difference more than 10mmHg between right and left arm systolic pressure and angiographic findings (irregular intimal surface, stenosis of the aorta or its branches, poststenotic dilatations, secular aneurysms or the typical narrowed "rat tail" appearance) [1] of hemodynamically significant lesions in the aorta or its major branches [3, 4, 7, 10], the presence of at least three of the above criteria confirms the diagnosis with 97% specificity and 92% sensitivity [1, 4]. Our patient is less than 40 years, has carotid bruit and a difference of more than 10mmHg in systolic blood pressure between right and left arm. Ishikawa et al, classified TA in pregnancy depending on the severity of retinopathy, secondary hypertension, aortic regurgitation and arterial aneurysm with particular indicators of maternal outcome: group I patients are with no complications, group IIa have one mild complication, group IIb have one severe complication and group III have several mild complications [2, 5-7]. Our patient having hypertension will be in group IIb. For group IIb and III, operative delivery is preferred with the aim of avoiding increased blood volume and hence arterial pressure which can occur during uterine contraction [5, 7] . Our patient being IIb and having oligohydramnios and IUGR operative delivery was planned. Preoperative evaluation involves identifying the distribution of affected arteries, degree of organ involvement with special attention to cardiac, pulmonary, renal and cerebral function in addition to drugs used for the treatment of TA [2]. Chronic use of corticosteroids could lead to suppression of endogenous corticosteroids release [7], hence our patient was given 100mg of hydrocortisone preoperatively. Invasive BP monitoring is advised in patients with BP measurement difficulty to obtain in any extremity and if rapid fluctuation in BP is anticipated [5-7, 10], which is not the case in our patient.

In addition to pregnancy induced physiological changes anaesthetic management in TA takes compromised regional circulation into consideration [8, 10, 11]. The anaesthetic goal in a patient with TA is the maintenance of blood pressure during perioperative period [11, 12]. Low dose regional anaesthesia (RA) combined with opioid causes less hemodynamic instability and allows easy monitoring of cerebral circulation [2, 10, 11]. Unlike general anaesthesia RA is associated with less risk of aspiration, pressure response during intubation and extubation which may aggravate hypertension and tachycardia leading to MI, Congestive heart failure (ccf) and intracranial hemorrhage [5, 7, 8, 11]. RA may cause hypotension inducing cerebral, renal, intestinal or uterine ischemia, [2, 6, 8, 10] but can be minimized by pre-anaesthetic volume expansion. Spinal anaesthesia hypotension can be corrected by generous IV fluid and by placing the patient in reverse Trendelenburg position [2]. Different doses of bupivacaine and fentanyl combination were reported with successful including 6.5 mg hyperbaric bupivacaine and 25 μg fentanyl [8]. We used 7.5mg of 0.5% hyperbaric bupivacaine combined with 20μg fentanyl successfully. Most TA patients need monitoring particularly for the first 24 hours following operative delivery under spinal anesthesia as about 60% of patients develop postoperative problems following inadequate control of arterial pressure including heart failure or fatal stroke in a small group of patients [5, 10]. Our patient's condition was optimal in recovery and she was transferred to the post-natal ward for regular BP monitoring.

## Conclusion

Blood pressure control during pregnancy, delivery and immediate post delivery period is an important step in reducing obstetric morbidity in this group of patients. Spinal anesthesia allows easy monitoring of cerebral perfusion. Although TA is very rare on the Africa continent, anesthesiologists should be up-to-date on the perioperative anesthetic management of TA.

## Competing interests

The authors declare no competing interests.
